# Supplementary figures and images for: Systematic Analysis of Long Non-Coding RNA Genes in Nonalcoholic Fatty Liver Disease
Source: Noncoding RNA. 2022 Jul 22;8(4):56. doi: 10.3390/ncrna8040056 (PMC9332188; doi:10.3390/ncrna8040056)

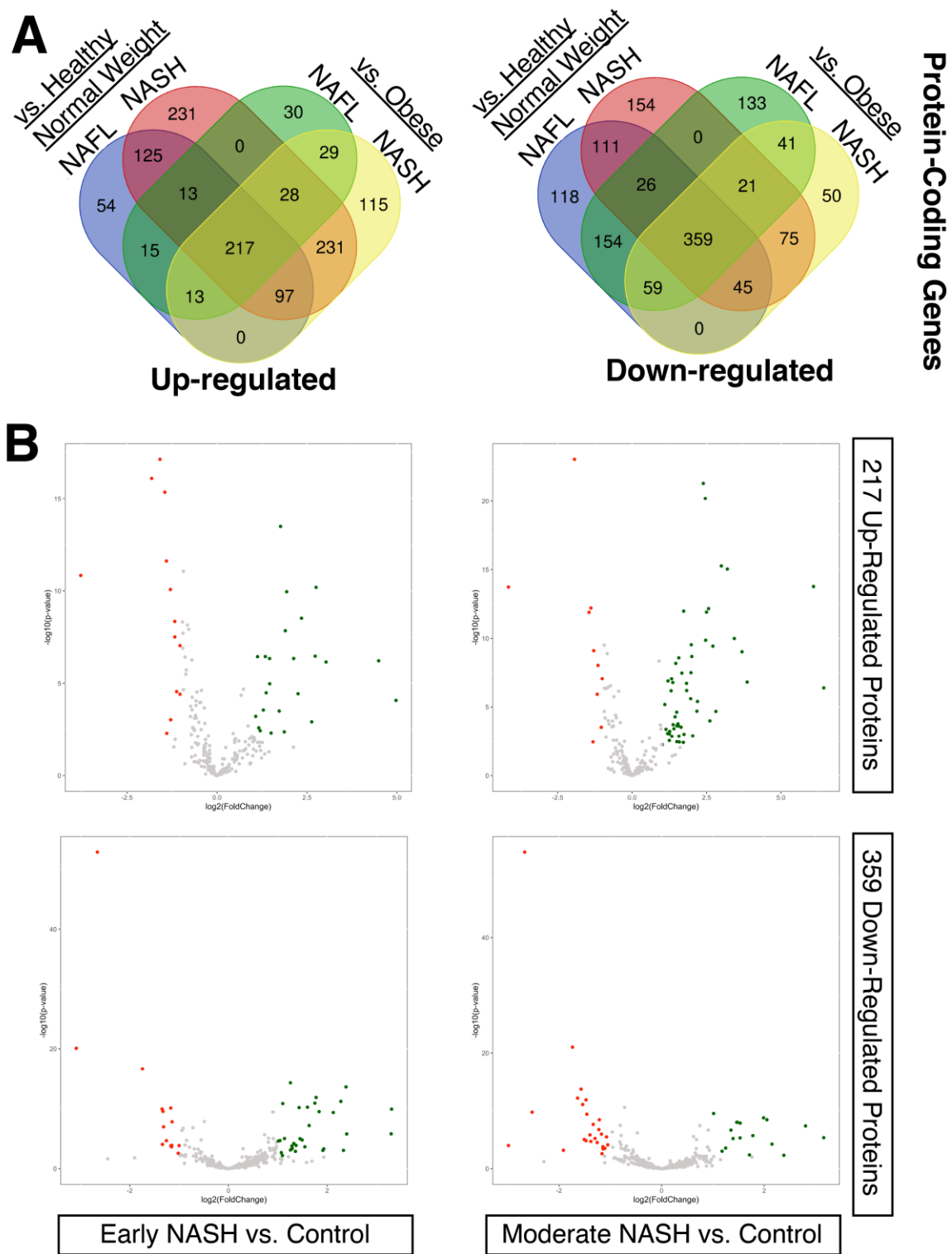

**Figure S1.** Differentially expressed protein-coding genes.

Supplement: Supplementary file 1 [file ncrna-08-00056-s001.zip › NAFLD-lncRNA-Supplementary_Figure_S1.pdf]
